# Supplementary material for: Physiological and Biochemical Responses of Almond (Prunus dulcis) Cultivars to Drought Stress in Semi-Arid Conditions in Iran
Source: Plants (Basel). 2025 Feb 27;14(5):734. doi: 10.3390/plants14050734 (PMC11902175; doi:10.3390/plants14050734)
Supplement: Supplementary file 1 [file plants-14-00734-s001.zip › plants-3450826-supplementary.pdf]

Supplemental information: Physiological and Biochemical Responses of Almond (Prunus dulcis) Cultivars to Drought Stress in Semi-Arid Conditions in Iran

Split plot RCBD - Statistical Summary

Anova Results

RWC MODEL - TWO YEARS

Table S1: Analysis of Variance RWC (continued below)

Source of Var.	Df	Sum sq	Mean sq	F value	P value
**RepxYear**	2	6.527	3.263	2	0.1379
**Year**	1	140.8	140.8	86.3	2.132e-17
**Rep**	2	9.057	4.529	2.775	0.06464
**Drought**	3	25459	8486	5201	2.813e-195
**DroughtxYear**	3	11.44	3.812	2.336	0.07482
**Error_a**	12	17.71	1.476		
**Cultivar**	13	5127	394.4	241.7	1.081e-117
**CultivarxYear**	13	9.613	0.7395	0.4532	0.9475
**DroughtxCultivar**	39	1063	27.25	16.7	2.041e-45
**DroughtxCultivarxYear**	39	22.14	0.5677	0.3479	0.9999
**Error_b**	208	339.4	1.632		
**Total**	335	32205			

Result
p = 0.138
p < .001
p = 0.065
p < .001
p = 0.075
p < .001
p = 0.948
p < .001
p > .999

```
## [1] "R Square 0.989"
```

```
## [1] "CV(a): 2.909 , CV(b) : 1.122"
```

Table S3: **Other Statistics**

	Standar Error of mean	Standar Error of difference	CD	CD1
Year	0.058	0.082	0.166	0.223
Rep	0.071	0.101	0.204	0.273
Drought	0.082	0.116	0.235	0.315
DroughtxYear	0.116	0.164	0.333	0.445
Cultivar	0.154	0.218	0.440	0.589
CultivarxYear	0.218	0.308	0.622	0.833
DroughtxCultivar	0.308	0.435	0.880	1.178
DroughtxCultivarxYear	0.435	0.615	1.244	1.666

Table S4: **Years Comparison**

Year	Means	Standard Error	Standard Dev.	Groups
1	67.773	0.766	9.930	a
2	66.479	0.746	9.664	b

Table S5: **Drought treatments Comparison**

Drought treatment	Means	Standard Error	Standard Dev.	Groups
10%	54.627	0.590	5.404	d
30%	63.658	0.513	4.699	c
50%	73.190	0.460	4.216	b
70%	77.029	0.381	3.495	a

Table S6: **Cultivars Comparison**

Cultivar	Means	Standard Error	Standard Dev.	Groups
Aidin	67.502	1.848	9.054	d
Araz	67.078	1.913	9.374	de
Eskandar	63.002	1.614	7.909	f
GN15(Garnem)	70.810	1.325	6.493	b
Mamaei	61.787	2.195	10.751	g
Rabie	63.171	2.262	11.080	f
Saba	67.377	1.991	9.753	d
Shahrood10	70.508	1.760	8.622	b
Shahrood12	66.083	1.509	7.393	e
Shahrood13	59.529	2.193	10.744	h
Shahrood21	68.691	2.008	9.837	c
Shahrood6	68.747	1.901	9.311	c
Shahrood7	72.330	1.995	9.773	a
Shahrood8	73.150	1.383	6.774	a

```
## The minimun for Drought treatments x Cultivars Comparison is: 10%:Shahrood13 = 45.86.
## The maximum is: 70%:Shahrood7 = 83.55.
```

Table S7: **Drought treatments x Cultivars Comparison**

DroughtxCultivar Interaction	Means	Standard Error	Standard Dev.	Groups
10%:Aidin	55.228	0.227	0.557	uv
10%:Araz	53.976	0.259	0.635	v
10%:Eskandar	53.875	1.448	3.547	v
10%:GN15(Garnem)	62.459	0.188	0.460	s
10%:Mamaei	45.864	0.344	0.842	w
10%:Rabie	48.198	0.362	0.886	w
10%:Saba	53.638	0.635	1.556	v
10%:Shahrood10	57.816	0.668	1.637	tu
10%:Shahrood12	55.161	0.216	0.529	uv
10%:Shahrood13	45.858	0.455	1.115	w
10%:Shahrood21	54.282	0.283	0.693	v
10%:Shahrood6	55.392	0.207	0.508	uv
10%:Shahrood7	58.678	0.904	2.215	t
10%:Shahrood8	64.357	0.228	0.558	qrs
30%:Aidin	63.070	0.509	1.247	rs
30%:Araz	63.280	0.431	1.055	qrs
30%:Eskandar	58.488	0.311	0.762	t
30%:GN15(Garnem)	67.274	0.261	0.639	nop
30%:Mamaei	59.018	0.287	0.704	t
30%:Rabie	58.320	0.322	0.789	t
30%:Saba	63.742	0.394	0.965	qrs
30%:Shahrood10	68.539	0.457	1.119	no
30%:Shahrood12	64.256	0.633	1.551	qrs
30%:Shahrood13	54.126	0.875	2.143	v
30%:Shahrood21	65.934	0.351	0.859	opq
30%:Shahrood6	65.992	0.340	0.833	opq
30%:Shahrood7	69.150	0.966	2.366	mn
30%:Shahrood8	70.025	0.515	1.262	klmn
50%:Aidin	75.032	0.365	0.895	efghi
50%:Araz	74.558	0.912	2.234	efghi
50%:Eskandar	65.967	0.577	1.413	opq
50%:GN15(Garnem)	75.123	0.335	0.819	efghi
50%:Mamaei	69.662	0.334	0.818	mn
50%:Rabie	69.900	0.493	1.208	lmn
50%:Saba	74.817	0.834	2.042	efghi
50%:Shahrood10	75.885	0.440	1.078	defgh
50%:Shahrood12	71.424	0.330	0.809	jklm
50%:Shahrood13	65.422	1.148	2.812	pqr
50%:Shahrood21	78.433	0.803	1.968	cd
50%:Shahrood6	74.099	0.336	0.824	fghij
50%:Shahrood7	77.941	0.617	1.511	cd
50%:Shahrood8	76.396	0.405	0.991	defg
70%:Aidin	76.680	0.404	0.989	def
70%:Araz	76.496	0.370	0.907	def
70%:Eskandar	73.679	0.369	0.903	ghij
70%:GN15(Garnem)	78.386	0.678	1.660	cd
70%:Mamaei	72.605	0.501	1.227	ijkl
70%:Rabie	76.265	0.870	2.131	defg
70%:Saba	77.309	0.693	1.698	cde
70%:Shahrood10	79.791	0.367	0.900	bc

DroughtxCultivar Interaction	Means	Standard Error	Standard Dev.	Groups
70%:Shahrood12	73.490	0.385	0.942	hij
70%:Shahrood13	72.712	1.132	2.773	ijk
70%:Shahrood21	76.117	0.532	1.304	defgh
70%:Shahrood6	79.505	0.349	0.855	bc
70%:Shahrood7	83.549	0.575	1.408	a
70%:Shahrood8	81.822	0.360	0.883	ab

ChlA MODEL - TWO YEARS

Table S8: Analysis of Variance ChlA

Source of Var.	Df	Sum sq	Mean sq	F value	P value	Result
**RepxYear**	2	1.393	0.6967	1.545	0.2157	p = 0.216
**Year**	1	31.82	31.82	70.56	6.945e-15	p < .001
**Rep**	2	0.0804	0.0402	0.08915	0.9147	p = 0.915
**Drought**	3	128.1	42.7	94.69	1.129e-38	p < .001
**DroughtxYear**	3	0.2928	0.09758	0.2164	0.8849	p = 0.885
**Error_a**	12	3.949	0.3291			
**Cultivar**	13	173.7	13.36	29.64	2.234e-40	p < .001
**CultivarxYear**	13	2.047	0.1575	0.3493	0.9828	p = 0.983
**DroughtxCultivar**	39	15.79	0.405	0.8981	0.6452	p = 0.645
**DroughtxCultivarxYear**	39	1.773	0.04547	0.1008	1	p > .999
**Error_b**	208	93.79	0.4509			
**Total**	335	452.8				

```
## [1] "R Square 0.793"
```

```
## [1] "CV(a): 4.563 , CV(b) : 3.115"
```

Table S9: **Other Statistics**

	Standar Error of mean	Standar Error of difference	CD	CD1
Year	0.058	0.082	0.166	0.223
Rep	0.071	0.101	0.204	0.273
Drought	0.082	0.116	0.235	0.315
DroughtxYear	0.116	0.164	0.333	0.445
Cultivar	0.154	0.218	0.440	0.589
CultivarxYear	0.218	0.308	0.622	0.833
DroughtxCultivar	0.308	0.435	0.880	1.178
DroughtxCultivarxYear	0.435	0.615	1.244	1.666

Table S10: **Years Comparison**

Year	Means	Standard Error	Standard Dev.	Groups
1	7.153	0.082	1.066	a
2	6.538	0.091	1.176	b

Table S11: **Drought treatments Comparison**

Drought treatment	Means	Standard Error	Standard Dev.	Groups
10%	5.912	0.120	1.101	d
30%	6.736	0.116	1.059	c
50%	7.149	0.105	0.964	b
70%	7.586	0.088	0.804	a

Table S12: **Cultivars Comparison**

Cultivar	Means	Standard Error	Standard Dev.	Groups
Aidin	7.210	0.175	0.857	bc
Araz	6.395	0.262	1.282	def
Eskandar	6.491	0.186	0.911	def
GN15(Garnem)	8.192	0.214	1.051	a
Mamaei	6.602	0.163	0.798	cdef
Rabie	6.464	0.226	1.106	def
Saba	5.982	0.148	0.725	f
Shahrood10	6.732	0.172	0.843	cd
Shahrood12	7.545	0.137	0.672	b
Shahrood13	6.624	0.213	1.041	cde
Shahrood21	6.355	0.172	0.841	def
Shahrood6	6.715	0.167	0.817	cd
Shahrood7	6.073	0.244	1.197	ef
Shahrood8	8.460	0.126	0.618	a

ChlB MODEL - TWO YEARS

Table S13: Analysis of Variance ChlB

Source of Var.	Df	Sum sq	Mean sq	F value	P value	Result
**RepxYear**	2	0.07752	0.03876	0.8201	0.4418	p = 0.442
**Year**	1	5.838	5.838	123.5	7.884e-23	p < .001
**Rep**	2	0.6436	0.3218	6.809	0.001367	p = 0.001
**Drought**	3	29.55	9.849	208.4	2.092e-62	p < .001
**DroughtxYear**	3	0.5323	0.1774	3.754	0.01175	p = 0.012
**Error_a**	12	0.5207	0.0434			
**Cultivar**	13	13.71	1.055	22.32	9.723e-33	p < .001
**CultivarxYear**	13	0.2316	0.01782	0.377	0.9758	p = 0.976
**DroughtxCultivar**	39	2.447	0.06275	1.328	0.1076	p = 0.108
**DroughtxCultivarxYear**	39	0.7471	0.01916	0.4053	0.9994	p > .999
**Error_b**	208	9.83	0.04726			
**Total**	335	64.13				

```
## [1] "R Square 0.847"
```

```
## [1] "CV(a): 16.632 , CV(b) : 5.465"
```

Table S14: **Other Statistics**

	Standar Error of mean	Standar Error of difference	CD	CD1
Year	0.058	0.082	0.166	0.223
Rep	0.071	0.101	0.204	0.273
Drought	0.082	0.116	0.235	0.315
DroughtxYear	0.116	0.164	0.333	0.445
Cultivar	0.154	0.218	0.440	0.589
CultivarxYear	0.218	0.308	0.622	0.833
DroughtxCultivar	0.308	0.435	0.880	1.178
DroughtxCultivarxYear	0.435	0.615	1.244	1.666

```
## The minimun for Repetitions x Year Comparison is: Rep2:Year2 = 2.36.
## The maximum is: Rep1:Year1 = 2.71.
```

Table S15: **Repetitions x Years Comparison**

RepetitionxYear Interaction	Means	Standard Error	Standard Dev.	Groups
Rep1:Year1	2.706	0.056	0.418	a
Rep1:Year2	2.466	0.050	0.378	b
Rep2:Year1	2.601	0.057	0.424	a
Rep2:Year2	2.356	0.049	0.367	c
Rep3:Year1	2.687	0.064	0.477	a
Rep3:Year2	2.380	0.058	0.433	bc

Table S16: **Repetitions Comparison**

Rep	Means	Standard Error	Standard Dev.	Groups
1	2.586	0.039	0.415	a
2	2.479	0.039	0.414	b
3	2.533	0.045	0.479	ab

Table S17: **Years Comparison**

Year	Means	Standard Error	Standard Dev.	Groups
1	2.664	0.034	0.440	a
2	2.401	0.030	0.394	b

Table S18: **Drought treatments Comparison**

Drought treatment	Means	Standard Error	Standard Dev.	Groups
10%	2.100	0.036	0.332	d
30%	2.443	0.031	0.282	c
50%	2.693	0.033	0.306	b
70%	2.894	0.040	0.365	a

```
## The minimum for Drought treatments x Years Comparison is: 10%:Year2 = 2.01.
## The maximum is: 70%:Year1 = 3.09.
```

Table S19: **Drought treatments x Years Comparison**

DroughtxYear Interaction	Means	Standard Error	Standard Dev.	Groups
10%:Year1	2.193	0.050	0.326	f
10%:Year2	2.007	0.049	0.315	g
30%:Year1	2.541	0.041	0.267	d
30%:Year2	2.344	0.041	0.263	e
50%:Year1	2.838	0.042	0.271	b
50%:Year2	2.549	0.042	0.272	d
70%:Year1	3.086	0.044	0.287	a
70%:Year2	2.703	0.052	0.335	c

Table S20: **Cultivars Comparison**

Cultivar	Means	Standard Error	Standard Dev.	Groups
Aidin	2.800	0.074	0.362	ab
Araz	2.335	0.086	0.419	ef
Eskandar	2.572	0.090	0.439	cd
GN15(Garnem)	2.813	0.075	0.366	a
Mamaei	2.569	0.094	0.460	cd
Rabie	2.565	0.099	0.487	cd
Saba	2.083	0.086	0.420	g
Shahrood10	2.598	0.062	0.302	bcd
Shahrood12	2.615	0.056	0.277	abcd
Shahrood13	2.348	0.091	0.446	ef
Shahrood21	2.264	0.087	0.424	fg
Shahrood6	2.719	0.085	0.417	abc
Shahrood7	2.493	0.084	0.411	de
Shahrood8	2.683	0.042	0.208	abcd

MDA MODEL - TWO YEARS

Table S21: Analysis of Variance MDA

Source of Var.	Df	Sum sq	Mean sq	F value	P value	Result
**RepxYear**	2	2.445	1.223	5.299	0.005692	p = 0.006
**Year**	1	88.96	88.96	385.6	2.976e-49	p < .001
**Rep**	2	1.061	0.5304	2.299	0.1029	p = 0.103
**Drought**	3	31616	10539	45677	6.964e-293	p < .001
**DroughtxYear**	3	31.63	10.54	45.7	9.965e-23	p < .001
**Error__a**	12	3.506	0.2921			
**Cultivar**	13	1634	125.7	544.8	9.831e-153	p < .001
**CultivarxYear**	13	1.034	0.07952	0.3447	0.9838	p = 0.984
**DroughtxCultivar**	39	1811	46.43	201.2	1.639e-144	p < .001
**DroughtxCultivarxYear**	39	5.585	0.1432	0.6206	0.9615	p = 0.961
**Error__b**	208	47.99	0.2307			

Source of Var.	Df	Sum sq	Mean sq	F value	P value	Result
**Total**	335	35243				

```
## [1] "R Square 0.999"
```

```
## [1] "CV(a): 14.704 , CV(b) : 1.714"
```

Table S22: **Other Statistics**

	Standar Error of mean	Standar Error of difference	CD	CD1
Year	0.058	0.082	0.166	0.223
Rep	0.071	0.101	0.204	0.273
Drought	0.082	0.116	0.235	0.315
DroughtxYear	0.116	0.164	0.333	0.445
Cultivar	0.154	0.218	0.440	0.589
CultivarxYear	0.218	0.308	0.622	0.833
DroughtxCultivar	0.308	0.435	0.880	1.178
DroughtxCultivarxYear	0.435	0.615	1.244	1.666

Table S23: **Years Comparison**

Year	Means	Standard Error	Standard Dev.	Groups
1	22.597	0.795	10.302	a
2	21.568	0.788	10.216	b

Table S24: **Drought treatments Comparison**

Drought treatment	Means	Standard Error	Standard Dev.	Groups
10%	38.198	0.651	5.969	a
30%	21.089	0.226	2.071	b
50%	15.337	0.150	1.377	c
70%	13.704	0.150	1.375	d

```
## The minimun for Drought treatments x Years Comparison  is:70%:Year2 = 13.69.
## The maximum is: 10%:Year1 = 38.76.
```

Table S25: **Drought treatments x Years Comparison**

DroughtxYear Interaction	Means	Standard Error	Standard Dev.	Groups
10%:Year1	38.759	0.896	5.809	a
10%:Year2	37.637	0.948	6.142	b
30%:Year1	21.743	0.310	2.010	c
30%:Year2	20.435	0.299	1.940	d
50%:Year1	16.170	0.167	1.080	e
50%:Year2	14.505	0.173	1.120	f

DroughtxYear Interaction	Means	Standard Error	Standard Dev.	Groups
70%:Year1	13.715	0.212	1.374	g
70%:Year2	13.694	0.215	1.392	g

Table S26: **Cultivars Comparison**

Cultivar	Means	Standard Error	Standard Dev.	Groups
Aidin	20.657	2.095	10.264	f
Araz	21.338	2.258	11.061	e
Eskandar	21.121	1.851	9.068	e
GN15(Garnem)	19.262	1.406	6.888	h
Mamaei	24.895	2.428	11.893	b
Rabie	24.185	2.596	12.716	c
Saba	22.847	2.226	10.907	d
Shahrood10	20.078	1.256	6.154	g
Shahrood12	19.317	1.502	7.358	h
Shahrood13	25.352	2.643	12.948	a
Shahrood21	25.354	2.214	10.847	a
Shahrood6	23.050	2.242	10.983	d
Shahrood7	22.672	2.424	11.873	d
Shahrood8	19.023	1.337	6.551	h

```
## The minimun for Drought treatments x Cultivars Comparison is:70%:Araz = 11.16.
## The maximum is: 10%:Shahrood13 = 46.64.
```

Table S27: **Drought treatments x Cultivar Comparison**

DroughtxCultivar Interaction	Means	Standard Error	Standard Dev.	Groups
10%:Aidin	37.448	0.619	1.517	g
10%:Araz	39.223	0.306	0.750	f
10%:Eskandar	35.938	0.306	0.750	h
10%:GN15(Garnem)	29.875	0.300	0.734	j
10%:Mamaei	43.870	0.320	0.785	c
10%:Rabie	45.092	0.631	1.547	b
10%:Saba	40.968	0.301	0.738	e
10%:Shahrood10	29.872	0.302	0.739	j
10%:Shahrood12	30.965	0.393	0.963	i
10%:Shahrood13	46.645	0.679	1.664	a
10%:Shahrood21	43.072	0.299	0.732	cd
10%:Shahrood6	40.212	0.325	0.795	ef
10%:Shahrood7	42.330	0.304	0.744	d
10%:Shahrood8	29.262	0.303	0.742	j
30%:Aidin	19.057	0.293	0.717	o
30%:Araz	20.145	0.272	0.666	n
30%:Eskandar	19.873	0.283	0.694	no
30%:GN15(Garnem)	20.010	0.316	0.774	no
30%:Mamaei	24.883	0.322	0.789	k
30%:Rabie	21.802	0.386	0.945	m
30%:Saba	20.020	0.275	0.674	no

DroughtxCultivar Interaction	Means	Standard Error	Standard Dev.	Groups
30%:Shahrood10	19.947	0.294	0.720	no
30%:Shahrood12	19.320	0.281	0.687	no
30%:Shahrood13	22.925	0.393	0.962	l
30%:Shahrood21	23.893	0.388	0.951	kl
30%:Shahrood6	24.033	0.296	0.726	k
30%:Shahrood7	20.000	0.323	0.792	no
30%:Shahrood8	19.335	0.272	0.665	no
50%:Aidin	13.692	0.402	0.984	vwx
50%:Araz	14.825	0.391	0.958	tu
50%:Eskandar	14.773	0.385	0.944	tu
50%:GN15(Garnem)	14.653	0.421	1.032	tuv
50%:Mamaei	15.932	0.385	0.942	qr
50%:Rabie	15.888	0.415	1.017	qrs
50%:Saba	15.935	0.382	0.937	qr
50%:Shahrood10	15.875	0.390	0.955	qrs
50%:Shahrood12	14.498	0.391	0.957	tuvw
50%:Shahrood13	16.702	0.455	1.114	q
50%:Shahrood21	17.808	0.420	1.029	p
50%:Shahrood6	14.522	0.393	0.964	tuvw
50%:Shahrood7	14.798	0.395	0.968	tu
50%:Shahrood8	14.822	0.391	0.958	tu
70%:Aidin	12.432	0.036	0.088	z
70%:Araz	11.158	0.048	0.117	A
70%:Eskandar	13.898	0.069	0.168	uvwx
70%:GN15(Garnem)	12.512	0.054	0.132	z
70%:Mamaei	14.895	0.037	0.090	stu
70%:Rabie	13.957	0.049	0.121	uvwx
70%:Saba	14.465	0.092	0.224	tuvw
70%:Shahrood10	14.617	0.151	0.369	tuv
70%:Shahrood12	12.485	0.069	0.170	z
70%:Shahrood13	15.138	0.113	0.277	rst
70%:Shahrood21	16.642	0.048	0.118	q
70%:Shahrood6	13.435	0.032	0.079	xyz
70%:Shahrood7	13.558	0.043	0.104	wxy
70%:Shahrood8	12.672	0.054	0.132	yz

EL MODEL - TWO YEARS

Table S28: Analysis of Variance EL

Source of Var.	Df	Sum sq	Mean sq	F value	P value	Result
**RepxYear**	2	150.1	75.03	35.08	7.461e-14	$p < .001$
**Year**	1	322.4	322.4	150.7	2.057e-26	$p < .001$
**Rep**	2	40.04	20.02	9.359	0.0001283	$p < .001$
**Drought**	3	65786	21929	10251	1.24e-225	$p < .001$
**DroughtxYear**	3	62	20.67	9.662	5.346e-06	$p < .001$
**Error_a**	12	266.9	22.24			
**Cultivar**	13	4575	351.9	164.5	1.088e-101	$p < .001$
**CultivarxYear**	13	0.5903	0.04541	0.02123	1	$p > .999$
**DroughtxCultivar**	39	3304	84.71	39.6	2.081e-76	$p < .001$
**DroughtxCultivarxYear**	39	7.644	0.196	0.09163	1	$p > .999$

Source of Var.	Df	Sum sq	Mean sq	F value	P value	Result
**Error__b**	208	444.9	2.139			
**Total**	335	74959				

```
## [1] "R Square 0.994"
```

```
## [1] "CV(a): 16.398 , CV(b) : 1.597"
```

Table S29: **Other Statistics**

	Standar Error of mean	Standar Error of difference	CD	CD1
Year	0.058	0.082	0.166	0.223
Rep	0.071	0.101	0.204	0.273
Drought	0.082	0.116	0.235	0.315
DroughtxYear	0.116	0.164	0.333	0.445
Cultivar	0.154	0.218	0.440	0.589
CultivarxYear	0.218	0.308	0.622	0.833
DroughtxCultivar	0.308	0.435	0.880	1.178
DroughtxCultivarxYear	0.435	0.615	1.244	1.666

```
## The minimun for Repetitions x Year Comparison is: Rep1:Year2 = 26.21.
## The maximum is: Rep2:Year1 = 29.89.
```

Table S30: **Repetitions x Years Comparison**

RepetitionxYear Interaction	Means	Standard Error	Standard Dev.	Groups
Rep1:Year1	28.454	1.994	14.924	b
Rep1:Year2	26.210	2.090	15.639	d
Rep2:Year1	29.889	1.871	14.001	a
Rep2:Year2	26.455	1.980	14.814	d
Rep3:Year1	27.767	2.082	15.580	c
Rep3:Year2	27.569	2.018	15.101	c

```
## [1] "Rep1" "Rep2" "Rep3"
```

Table S31: **Repetitions Comparison**

Rep	Means	Standard Error	Standard Dev.	Groups
1	27.332	1.442	15.259	b
2	28.172	1.366	14.451	a
3	27.668	1.443	15.273	b

Table S32: **Years Comparison**

Year	Means	Standard Error	Standard Dev.	Groups
1	28.703	1.141	14.787	a
2	26.744	1.166	15.109	b

Table S33: **Drought treatments Comparison**

Drought treatment	Means	Standard Error	Standard Dev.	Groups
10%	46.788	0.795	7.288	a
30%	35.404	0.724	6.639	b
50%	15.609	0.362	3.315	c
70%	13.096	0.167	1.529	d

```
## The minimun for Drought treatments x Years Comparison  is: 70%:Year2 = 12.46.
## The maximum is: 10%:Year1 = 47.41.
```

Table S34: **Drought treatments x Years Comparison**

DroughtxYear Interaction	Means	Standard Error	Standard Dev.	Groups
10%:Year1	47.414	1.104	7.153	a
10%:Year2	46.161	1.150	7.454	b
30%:Year1	36.376	1.024	6.635	c
30%:Year2	34.431	1.015	6.578	d
50%:Year1	17.292	0.513	3.322	e
50%:Year2	13.925	0.359	2.325	f
70%:Year1	13.732	0.239	1.547	f
70%:Year2	12.460	0.190	1.230	g

Table S35: **Cultivars Comparison**

Cultivar	Means	Standard Error	Standard Dev.	Groups
Aidin	24.365	2.613	12.800	f
Araz	26.348	2.554	12.512	e
Eskandar	26.506	2.695	13.203	e
GN15(Garnem)	23.019	2.283	11.186	g
Mamaei	32.069	3.690	18.077	b
Rabie	31.083	3.606	17.666	b
Saba	28.182	3.077	15.074	d
Shahrood10	24.536	2.618	12.824	f
Shahrood12	24.377	2.131	10.442	f
Shahrood13	37.233	4.278	20.956	a
Shahrood21	29.745	3.440	16.852	c
Shahrood6	27.872	3.033	14.859	d
Shahrood7	27.824	3.021	14.798	d
Shahrood8	24.976	2.397	11.743	f

```
## The minimun for Drought treatments x Genoytpes Comparison is: 70%:Aidin = 10.24.
## The maximum is: 10%:Shahrood13 = 61.22.
```

Table S36: **Drought treatments x Cultivars Comparison**

DroughtxCultivar Interaction	Means	Standard Error	Standard Dev.	Groups
10%:Aidin	39.513	0.406	0.995	fg
10%:Araz	44.190	0.367	0.900	e
10%:Eskandar	43.297	0.425	1.041	e
10%:GN15(Garnem)	37.843	0.389	0.952	gh
10%:Mamaei	54.167	0.392	0.961	bc
10%:Rabie	56.078	0.867	2.124	b
10%:Saba	48.570	0.467	1.145	d
10%:Shahrood10	42.438	0.376	0.921	ef
10%:Shahrood12	38.217	0.417	1.021	gh
10%:Shahrood13	61.223	0.536	1.313	a
10%:Shahrood21	53.617	0.381	0.933	bc
10%:Shahrood6	49.147	0.448	1.097	d
10%:Shahrood7	48.197	0.498	1.220	d
10%:Shahrood8	38.532	0.479	1.173	gh
30%:Aidin	33.530	0.678	1.660	jk
30%:Araz	30.877	0.544	1.333	kl
30%:Eskandar	34.550	0.547	1.340	ij
30%:GN15(Garnem)	28.877	0.544	1.333	l
30%:Mamaei	44.507	0.634	1.554	e
30%:Rabie	37.582	0.663	1.625	ghi
30%:Saba	35.632	0.620	1.519	hij
30%:Shahrood10	29.830	0.561	1.374	l
30%:Shahrood12	29.735	0.639	1.565	l
30%:Shahrood13	52.698	3.416	8.367	c
30%:Shahrood21	36.292	0.855	2.095	ghij
30%:Shahrood6	33.230	0.538	1.318	jk
30%:Shahrood7	34.445	0.647	1.585	ij
30%:Shahrood8	33.867	0.567	1.388	jk
50%:Aidin	14.177	1.215	2.976	opq
50%:Araz	15.973	1.171	2.868	no
50%:Eskandar	15.027	1.284	3.146	nopq
50%:GN15(Garnem)	13.097	1.291	3.162	opqr
50%:Mamaei	15.877	1.228	3.009	no
50%:Rabie	18.203	1.217	2.981	mn
50%:Saba	15.153	1.180	2.891	nop
50%:Shahrood10	14.063	1.208	2.958	opq
50%:Shahrood12	15.853	1.224	2.998	no
50%:Shahrood13	20.863	1.212	2.969	m
50%:Shahrood21	15.190	1.213	2.970	nop
50%:Shahrood6	14.077	1.171	2.870	opq
50%:Shahrood7	15.890	1.207	2.956	no
50%:Shahrood8	15.077	1.171	2.870	nop
70%:Aidin	10.240	0.383	0.939	r
70%:Araz	14.351	0.372	0.912	opq
70%:Eskandar	13.150	0.389	0.953	opqr
70%:GN15(Garnem)	12.259	0.386	0.946	pqr
70%:Mamaei	13.728	0.394	0.964	opq
70%:Rabie	12.468	0.840	2.057	pqr
70%:Saba	13.372	0.385	0.944	opqr
70%:Shahrood10	11.813	0.400	0.979	qr

DroughtxCultivar Interaction	Means	Standard Error	Standard Dev.	Groups
70%:Shahrood12	13.702	0.380	0.931	opq
70%:Shahrood13	14.148	0.370	0.906	opq
70%:Shahrood21	13.883	0.375	0.919	opq
70%:Shahrood6	15.036	0.372	0.912	nopq
70%:Shahrood7	12.765	0.375	0.918	opqr
70%:Shahrood8	12.428	0.379	0.929	pqr

Proline MODEL - TWO YEARS

Table S37: Analysis of Variance Proline

Source of Var.	Df	Sum sq	Mean sq	F value	P value	Result
**RepxYear**	2	79.8	39.9	7.294	0.000868	p < .001
**Year**	1	184.8	184.8	33.77	2.301e-08	p < .001
**Rep**	2	112.7	56.35	10.3	5.427e-05	p < .001
**Drought**	3	51865	17288	3160	3.654e-173	p < .001
**DroughtxYear**	3	4.08	1.36	0.2486	0.8623	p = 0.862
**Error_a**	12	137.4	11.45			
**Cultivar**	13	2184	168	30.71	2.111e-41	p < .001
**CultivarxYear**	13	44.98	3.46	0.6325	0.8252	p = 0.825
**DroughtxCultivar**	39	2156	55.28	10.11	1.634e-30	p < .001
**DroughtxCultivarxYear**	39	135	3.462	0.6328	0.9552	p = 0.955
**Error_b**	208	1138	5.47			
**Total**	335	58042				

```
## [1] "R Square 0.98"
```

```
## [1] "CV(a): 1.569 , CV(b) : 2.503"
```

Table S38: **Other Statistics**

	Standar Error of mean	Standar Error of difference	CD	CD1
Year	0.058	0.082	0.166	0.223
Rep	0.071	0.101	0.204	0.273
Drought	0.082	0.116	0.235	0.315
DroughtxYear	0.116	0.164	0.333	0.445
Cultivar	0.154	0.218	0.440	0.589
CultivarxYear	0.218	0.308	0.622	0.833
DroughtxCultivar	0.308	0.435	0.880	1.178
DroughtxCultivarxYear	0.435	0.615	1.244	1.666

```
## The minimun for Repetitions x Year Comparison is: Rep2:Year1 = 30.77.
## The maximum is: Rep3:Year2 = 34.24.
```

Table S39: **Repetitions x Years Comparison**

RepetitionxYear Interaction	Means	Standard Error	Standard Dev.	Groups
Rep1:Year1	31.905	1.751	13.105	bc
Rep1:Year2	32.017	1.699	12.712	b
Rep2:Year1	30.770	1.729	12.942	c
Rep2:Year2	32.813	1.665	12.462	b
Rep3:Year1	31.949	1.731	12.954	bc
Rep3:Year2	34.243	2.002	14.984	a

Table S40: **Repetitions Comparison**

Rep	Means	Standard Error	Standard Dev.	Groups
1	31.961	1.214	12.852	b
2	31.792	1.199	12.688	b
3	33.096	1.322	13.990	a

Table S41: **Years Comparison**

Year	Means	Standard Error	Standard Dev.	Groups
1	31.541	0.998	12.934	b
2	33.024	1.033	13.385	a

Table S42: **Drought treatments Comparison**

Drought treatment	Means	Standard Error	Standard Dev.	Groups
10%	52.002	0.733	6.718	a
30%	33.252	0.384	3.518	b
50%	24.554	0.304	2.784	c
70%	19.324	0.330	3.027	d

Table S43: **Cultivars Comparison**

Cultivar	Means	Standard Error	Standard Dev.	Groups
Aidin	31.852	2.724	13.345	de
Araz	32.341	2.896	14.187	cde
Eskandar	33.044	2.880	14.107	bcd
GN15(Garnem)	33.854	2.301	11.273	bcd
Mamaei	29.222	2.538	12.432	fg
Rabie	28.233	1.883	9.227	g
Saba	30.634	2.685	13.156	ef
Shahrood10	34.176	2.584	12.659	bc
Shahrood12	33.319	2.172	10.639	bcd
Shahrood13	35.198	3.521	17.250	b
Shahrood21	28.464	2.277	11.156	fg
Shahrood6	37.451	3.005	14.721	a

Cultivar	Means	Standard Error	Standard Dev.	Groups
Shahrood7	30.543	2.774	13.591	ef
Shahrood8	33.627	2.983	14.613	bcd

```
## The minimun for Drought treatments x Genoytpes Comparison is: 70%:Saba = 15.11.
## The maximum is: 10%:Shahrood6 = 61.
```

Table S44: **Drought treatments x Years Comparison**

DroughtxCultivar Interaction	Means	Standard Error	Standard Dev.	Groups
10%:Aidin	52.974	0.637	1.560	bcd
10%:Araz	51.758	0.250	0.612	cd
10%:Eskandar	54.522	0.486	1.190	bc
10%:GN15(Garnem)	50.614	0.263	0.645	cde
10%:Mamaei	49.778	0.927	2.271	cde
10%:Rabie	41.267	1.866	4.570	fg
10%:Saba	48.447	0.559	1.369	de
10%:Shahrood10	52.665	0.639	1.565	bcd
10%:Shahrood12	49.804	0.449	1.100	cde
10%:Shahrood13	60.697	6.267	15.350	a
10%:Shahrood21	45.560	0.611	1.498	ef
10%:Shahrood6	60.997	0.467	1.145	a
10%:Shahrood7	51.189	1.741	4.264	cd
10%:Shahrood8	57.752	0.303	0.743	ab
30%:Aidin	31.192	0.316	0.774	jklmn
30%:Araz	38.334	0.531	1.300	gh
30%:Eskandar	34.284	0.538	1.319	hijkl
30%:GN15(Garnem)	35.657	0.597	1.462	hijk
30%:Mamaei	26.147	0.380	0.930	nopqrst
30%:Rabie	30.472	0.477	1.168	klmno
30%:Saba	36.487	0.505	1.237	ghi
30%:Shahrood10	37.254	0.429	1.051	ghi
30%:Shahrood12	34.270	0.339	0.829	hijkl
30%:Shahrood13	33.442	0.377	0.924	hijkl
30%:Shahrood21	29.170	0.317	0.776	lmnop
30%:Shahrood6	36.240	0.468	1.146	ghij
30%:Shahrood7	32.114	0.340	0.834	ijklm
30%:Shahrood8	30.464	0.488	1.195	klmno
50%:Aidin	24.977	0.364	0.892	pqrstu
50%:Araz	23.645	0.371	0.909	qrstuv
50%:Eskandar	25.738	0.384	0.939	opqrst
50%:GN15(Garnem)	28.214	0.487	1.193	mnopq
50%:Mamaei	19.904	0.365	0.894	uvwxyzA
50%:Rabie	21.874	1.196	2.930	rstuvwxy
50%:Saba	22.488	0.370	0.906	rstuvwx
50%:Shahrood10	26.997	0.357	0.876	mnopqr
50%:Shahrood12	26.279	0.420	1.029	nopqrs
50%:Shahrood13	25.155	0.438	1.073	pqrst
50%:Shahrood21	22.840	0.367	0.899	rstuvwx
50%:Shahrood6	29.182	0.475	1.163	lmnop
50%:Shahrood7	22.160	0.475	1.164	rstuvwx

DroughtxCultivar Interaction	Means	Standard Error	Standard Dev.	Groups
50%:Shahrood8	24.307	0.589	1.443	pqrstuv
70%:Aidin	18.265	0.451	1.105	wxyzA
70%:Araz	15.627	0.489	1.197	A
70%:Eskandar	17.631	1.073	2.629	xyzA
70%:GN15(Garnem)	20.934	0.427	1.046	tuvwxyz
70%:Mamaei	21.060	0.516	1.265	tuvwxyz
70%:Rabie	19.322	1.089	2.666	vwxyzA
70%:Saba	15.112	0.464	1.136	A
70%:Shahrood10	19.790	0.411	1.006	uvwxyzA
70%:Shahrood12	22.925	0.478	1.172	rstuvw
70%:Shahrood13	21.498	0.389	0.952	stuvwxyz
70%:Shahrood21	16.285	0.508	1.246	zA
70%:Shahrood6	23.384	1.026	2.514	qrstuvw
70%:Shahrood7	16.709	0.413	1.012	yzA
70%:Shahrood8	21.987	0.413	1.011	rstuvwx

Soluble Carbohydrates MODEL - TWO YEARS

Table S45: Analysis of Variance Soluble Carbohydrates

Source of Var.	Df	Sum sq	Mean sq	F value	P value	Result
**RepxYear**	2	19.01	9.504	10.61	4.103e-05	p < .001
**Year**	1	212.7	212.7	237.4	3.042e-36	p < .001
**Rep**	2	9.659	4.829	5.39	0.00522	p = 0.005
**Drought**	3	47015	15672	17492	1.218e-249	p < .001
**DroughtxYear**	3	17.94	5.979	6.674	0.0002531	p < .001
**Error__a**	12	56.76	4.73			
**Cultivar**	13	11148	857.6	957.2	1.334e-177	p < .001
**CultivarxYear**	13	3.924	0.3019	0.3369	0.9854	p = 0.985
**DroughtxCultivar**	39	3090	79.23	88.44	4.266e-109	p < .001
**DroughtxCultivarxYear**	39	8.611	0.2208	0.2464	1	p > .999
**Error__b**	208	186.4	0.8959			
**Total**	335	61768				

```
## [1] "R Square 0.997"
```

```
## [1] "CV(a): 3.29 , CV(b) : 0.632"
```

Table S46: **Other Statistics**

	Standar Error of mean	Standar Error of difference	CD	CD1
Year	0.058	0.082	0.166	0.223
Rep	0.071	0.101	0.204	0.273
Drought	0.082	0.116	0.235	0.315
DroughtxYear	0.116	0.164	0.333	0.445
Cultivar	0.154	0.218	0.440	0.589
CultivarxYear	0.218	0.308	0.622	0.833
DroughtxCultivar	0.308	0.435	0.880	1.178

	Standar Error of mean	Standar Error of difference	CD	CD1
DroughtxCultivarxYear	0.435	0.615	1.244	1.666

```
## The minimun for Repetitions x Year Comparison is: Rep2:Year1 = 73.06.
## The maximum is: Rep3:Year2 = 75.37.
```

Table S47: **Repetitions x Years Comparison**

RepetitionxYear Interaction	Means	Standard Error	Standard Dev.	Groups
Rep1:Year1	73.875	1.930	14.444	c
Rep1:Year2	74.843	1.801	13.478	b
Rep2:Year1	73.056	1.776	13.292	d
Rep2:Year2	75.179	1.761	13.178	ab
Rep3:Year1	73.689	1.843	13.789	c
Rep3:Year2	75.372	1.832	13.708	a

Table S48: **Repetitions Comparison**

Rep	Means	Standard Error	Standard Dev.	Groups
1	74.359	1.315	13.915	ab
2	74.117	1.249	13.218	b
3	74.530	1.296	13.713	a

Table S49: **Years Comparison**

Year	Means	Standard Error	Standard Dev.	Groups
1	73.540	1.062	13.771	b
2	75.131	1.032	13.377	a

Table S50: **Drought treatments Comparison**

Drought treatment	Means	Standard Error	Standard Dev.	Groups
10%	89.098	0.944	8.654	a
30%	79.473	0.546	5.002	b
50%	72.047	0.616	5.647	c
70%	56.724	0.740	6.778	d

Table S51: **Cultivars Comparison**

Cultivar	Means	Standard Error	Standard Dev.	Groups
Aidin	78.088	2.673	13.093	d
Araz	67.853	2.626	12.863	j
Eskandar	71.673	2.747	13.457	g
GN15(Garnem)	84.673	2.812	13.777	a

Cultivar	Means	Standard Error	Standard Dev.	Groups
Mamaei	72.422	2.050	10.041	f
Rabie	75.388	2.282	11.180	e
Saba	68.031	2.791	13.674	j
Shahrood10	78.459	2.569	12.585	d
Shahrood12	83.889	2.921	14.309	b
Shahrood13	68.557	2.397	11.745	ij
Shahrood21	72.028	2.034	9.966	fg
Shahrood6	68.778	2.355	11.539	i
Shahrood7	69.528	2.472	12.109	h
Shahrood8	81.331	2.888	14.147	c

```
## The minimun for Drought treatments x Genoytpes Comparison is: 70%:Araz = 47.82.
## The maximum is: 10%:GN15(Garnem) = 105.16.
```

Table S52: **Drought treatments x Years Comparison**

DroughtxCultivar Interaction	Means	Standard Error	Standard Dev.	Groups
10%:Aidin	90.467	0.411	1.006	cd
10%:Araz	80.445	0.729	1.786	jklm
10%:Eskandar	84.955	0.522	1.279	efg
10%:GN15(Garnem)	105.160	0.432	1.059	a
10%:Mamaei	86.543	0.398	0.975	e
10%:Rabie	88.890	0.692	1.695	d
10%:Saba	81.188	0.523	1.281	ijk
10%:Shahrood10	90.908	0.377	0.923	c
10%:Shahrood12	103.025	1.057	2.588	b
10%:Shahrood13	84.040	1.540	3.773	fgh
10%:Shahrood21	83.803	0.362	0.887	fgh
10%:Shahrood6	80.755	0.483	1.182	jkl
10%:Shahrood7	83.048	0.383	0.938	ghi
10%:Shahrood8	104.138	0.387	0.949	ab
30%:Aidin	85.202	0.652	1.596	ef
30%:Araz	76.088	0.359	0.879	o
30%:Eskandar	79.738	0.316	0.774	klmn
30%:GN15(Garnem)	86.432	0.675	1.653	e
30%:Mamaei	74.578	0.321	0.785	op
30%:Rabie	82.142	0.321	0.786	hij
30%:Saba	79.298	0.319	0.783	klmn
30%:Shahrood10	86.073	0.361	0.885	e
30%:Shahrood12	86.758	0.479	1.172	e
30%:Shahrood13	72.498	0.326	0.799	qr
30%:Shahrood21	75.555	0.445	1.089	op
30%:Shahrood6	74.995	0.346	0.848	op
30%:Shahrood7	74.075	0.356	0.872	pq
30%:Shahrood8	79.188	0.365	0.893	lmn
50%:Aidin	79.682	0.468	1.146	klmn
50%:Araz	67.057	0.389	0.952	w
50%:Eskandar	71.505	0.413	1.012	rst
50%:GN15(Garnem)	78.637	0.446	1.092	mn
50%:Mamaei	69.100	0.408	0.999	uv

DroughtxCultivar Interaction	Means	Standard Error	Standard Dev.	Groups
50%:Rabie	69.685	0.515	1.262	tu
50%:Saba	63.375	0.400	0.980	x
50%:Shahrood10	78.162	0.390	0.955	n
50%:Shahrood12	81.815	0.407	0.996	ij
50%:Shahrood13	64.515	0.443	1.085	x
50%:Shahrood21	71.738	0.466	1.142	rs
50%:Shahrood6	68.560	0.520	1.274	uvw
50%:Shahrood7	70.292	0.393	0.964	stu
50%:Shahrood8	74.538	0.490	1.200	op
70%:Aidin	57.002	0.648	1.588	A
70%:Araz	47.822	0.618	1.513	D
70%:Eskandar	50.495	0.533	1.305	C
70%:GN15(Garnem)	68.465	0.539	1.320	uvw
70%:Mamaei	59.465	0.523	1.281	yz
70%:Rabie	60.835	0.737	1.805	y
70%:Saba	48.262	0.547	1.339	D
70%:Shahrood10	58.693	0.498	1.220	zA
70%:Shahrood12	63.958	0.519	1.271	x
70%:Shahrood13	53.173	1.194	2.924	B
70%:Shahrood21	57.015	0.529	1.295	A
70%:Shahrood6	50.802	0.627	1.536	C
70%:Shahrood7	50.698	0.507	1.243	C
70%:Shahrood8	67.458	0.502	1.230	vw

Total Phenol MODEL - TWO YEARS

Table S53: Analysis of Variance Total Phenol

Source of Var.	Df	Sum sq	Mean sq	F value	P value	Result
**RepxYear**	2	59.44	29.72	174.9	2.768e-45	p < .001
**Year**	1	873.2	873.2	5140	1.261e-148	p < .001
**Rep**	2	18.81	9.407	55.37	5.276e-20	p < .001
**Drought**	3	18326	6109	35953	4.328e-282	p < .001
**DroughtxYear**	3	1.023	0.3411	2.008	0.114	p = 0.114
**Error_a**	12	6.697	0.5581			
**Cultivar**	13	1992	153.2	901.9	5.832e-175	p < .001
**CultivarxYear**	13	0.216	0.01662	0.09779	1	p > .999
**DroughtxCultivar**	39	1442	36.97	217.6	6.165e-148	p < .001
**DroughtxCultivarxYear**	39	1.905	0.04884	0.2874	1	p > .999
**Error_b**	208	35.34	0.1699			
**Total**	335	22756				

```
## [1] "R Square 0.998"
```

```
## [1] "CV(a): 2.287 , CV(b) : 0.865"
```

Table S54: **Other Statistics**

	Standar Error of mean	Standar Error of difference	CD	CD1
Year	0.058	0.082	0.166	0.223
Rep	0.071	0.101	0.204	0.273
Drought	0.082	0.116	0.235	0.315
DroughtxYear	0.116	0.164	0.333	0.445
Cultivar	0.154	0.218	0.440	0.589
CultivarxYear	0.218	0.308	0.622	0.833
DroughtxCultivar	0.308	0.435	0.880	1.178
DroughtxCultivarxYear	0.435	0.615	1.244	1.666

```
## The minimun for Repetitions x Year Comparison is: Rep3:Year1 = 23.74.
## The maximum is: Rep2:Year2 = 27.89.
```

Table S55: **Repetitions x Years Comparison**

RepetitionxYear Interaction	Means	Standard Error	Standard Dev.	Groups
Rep1:Year1	24.229	1.061	7.943	d
Rep1:Year2	26.314	1.093	8.182	c
Rep2:Year1	23.801	1.076	8.054	e
Rep2:Year2	27.891	1.082	8.097	a
Rep3:Year1	23.744	1.113	8.328	e
Rep3:Year2	27.242	1.091	8.162	b

Table S56: **Repetitions Comparison**

Rep	Means	Standard Error	Standard Dev.	Groups
1	25.271	0.765	8.095	c
2	25.846	0.784	8.297	a
3	25.493	0.793	8.394	b

Table S57: **Years Comparison**

Year	Means	Standard Error	Standard Dev.	Groups
1	23.925	0.622	8.064	b
2	27.149	0.627	8.124	a

Table S58: **Drought treatments Comparison**

Drought treatment	Means	Standard Error	Standard Dev.	Groups
10%	35.765	0.564	5.172	a
30%	28.424	0.349	3.196	b
50%	22.079	0.348	3.193	c
70%	15.880	0.272	2.493	d

Table S59: **Cultivars Comparison**

Cultivar	Means	Standard Error	Standard Dev.	Groups
Aidin	24.879	1.512	7.408	h
Araz	25.144	1.481	7.255	gh
Eskandar	25.305	1.373	6.724	g
GN15(Garnem)	29.337	1.803	8.832	b
Mamaei	26.492	2.209	10.823	e
Rabie	22.431	1.285	6.297	j
Saba	22.166	1.516	7.427	j
Shahrood10	27.285	1.388	6.800	d
Shahrood12	29.835	2.001	9.801	a
Shahrood13	23.005	1.453	7.119	i
Shahrood21	22.343	1.414	6.928	j
Shahrood6	25.862	1.441	7.061	f
Shahrood7	25.139	1.721	8.429	gh
Shahrood8	28.293	2.023	9.911	c

```
## The minimun for Drought treatments x Genoytpes Comparison is: 70%:Saba = 12.46.
## The maximum is: 10%:Mamaei = 43.86.
```

Table S60: **Drought treatments x Cultivars Comparison**

DroughtxCultivar Interaction	Means	Standard Error	Standard Dev.	Groups
10%:Aidin	33.085	0.771	1.889	def
10%:Araz	33.183	0.713	1.746	def
10%:Eskandar	33.602	0.771	1.888	d
10%:GN15(Garnem)	41.053	0.729	1.786	b
10%:Mamaei	43.862	0.761	1.865	a
10%:Rabie	30.108	1.016	2.489	hi
10%:Saba	32.108	0.791	1.938	g
10%:Shahrood10	35.108	0.767	1.878	c
10%:Shahrood12	43.528	0.763	1.869	a
10%:Shahrood13	32.832	0.923	2.261	defg
10%:Shahrood21	29.792	0.758	1.856	i
10%:Shahrood6	33.425	0.780	1.912	de
10%:Shahrood7	35.702	0.861	2.110	c
10%:Shahrood8	43.322	0.777	1.904	a
30%:Aidin	28.717	0.703	1.722	j
30%:Araz	29.648	0.728	1.782	i
30%:Eskandar	27.065	0.715	1.752	kl
30%:GN15(Garnem)	32.588	0.716	1.753	fg
30%:Mamaei	24.982	0.782	1.914	no
30%:Rabie	25.318	0.737	1.805	no
30%:Saba	23.792	0.706	1.729	p
30%:Shahrood10	30.215	0.737	1.805	hi
30%:Shahrood12	32.692	0.707	1.732	efg
30%:Shahrood13	25.475	0.717	1.757	mno
30%:Shahrood21	27.118	0.723	1.772	k
30%:Shahrood6	30.675	0.718	1.759	h
30%:Shahrood7	30.068	0.717	1.756	hi

DroughtxCultivar Interaction	Means	Standard Error	Standard Dev.	Groups
30%:Shahrood8	29.578	0.724	1.774	i
50%:Aidin	23.552	0.818	2.004	p
50%:Araz	22.572	0.749	1.835	q
50%:Eskandar	24.728	0.755	1.850	o
50%:GN15(Garnem)	25.612	0.759	1.859	mn
50%:Mamaei	20.502	0.768	1.881	s
50%:Rabie	19.935	0.777	1.903	st
50%:Saba	20.308	0.843	2.065	s
50%:Shahrood10	26.265	0.798	1.954	lm
50%:Shahrood12	25.045	0.779	1.907	no
50%:Shahrood13	17.692	1.028	2.518	w
50%:Shahrood21	19.348	0.755	1.850	tu
50%:Shahrood6	23.052	0.769	1.885	pq
50%:Shahrood7	18.888	0.748	1.832	uv
50%:Shahrood8	21.605	0.781	1.912	r
70%:Aidin	14.163	0.767	1.880	A
70%:Araz	15.173	0.749	1.836	yz
70%:Eskandar	15.823	0.745	1.824	xy
70%:GN15(Garnem)	18.097	0.751	1.839	vw
70%:Mamaei	16.623	0.758	1.857	x
70%:Rabie	14.363	0.709	1.738	zA
70%:Saba	12.457	0.784	1.919	B
70%:Shahrood10	17.550	0.750	1.838	w
70%:Shahrood12	18.073	0.757	1.853	vw
70%:Shahrood13	16.023	0.712	1.745	x
70%:Shahrood21	13.113	0.798	1.955	B
70%:Shahrood6	16.297	0.743	1.820	x
70%:Shahrood7	15.897	0.762	1.866	xy
70%:Shahrood8	18.667	0.766	1.876	uv

CAT MODEL - TWO YEARS

Table S61: Analysis of Variance Total CAT

Source of Var.	Df	Sum sq	Mean sq	F value	P value	Result
**RepxYear**	2	1.239	0.6194	12.67	6.434e-06	p < .001
**Year**	1	98.33	98.33	2011	6.947e-109	p < .001
**Rep**	2	0.7141	0.3571	7.302	0.000861	p < .001
**Drought**	3	4607	1536	31406	5.383e-276	p < .001
**DroughtxYear**	3	8.002	2.667	54.55	4.698e-26	p < .001
**Error_a**	12	4.272	0.356			
**Cultivar**	13	1093	84.06	1719	1.056e-203	p < .001
**CultivarxYear**	13	0.2241	0.01724	0.3526	0.9821	p = 0.982
**DroughtxCultivar**	39	624.2	16	327.3	6.015e-166	p < .001
**DroughtxCultivarxYear**	39	0.8707	0.02233	0.4566	0.9977	p = 0.998
**Error_b**	208	10.17	0.0489			
**Total**	335	6448				

```
## [1] "R Square 0.998"
```

```
## [1] "CV(a): 11.351 , CV(b) : 1.038"
```

Table S62: **Other Statistics**

	Standar Error of mean	Standar Error of difference	CD	CD1
Year	0.058	0.082	0.166	0.223
Rep	0.071	0.101	0.204	0.273
Drought	0.082	0.116	0.235	0.315
DroughtxYear	0.116	0.164	0.333	0.445
Cultivar	0.154	0.218	0.440	0.589
CultivarxYear	0.218	0.308	0.622	0.833
DroughtxCultivar	0.308	0.435	0.880	1.178
DroughtxCultivarxYear	0.435	0.615	1.244	1.666

```
## The minimun for Repetitions x Year Comparison is: Rep2:Year1 = 13.83.
## The maximum is: Rep2:Year2 = 15.08.
```

Table S63: **Repetitions x Years Comparison**

RepetitionxYear Interaction	Means	Standard Error	Standard Dev.	Groups
Rep1:Year1	13.848	0.566	4.238	c
Rep1:Year2	14.872	0.584	4.369	b
Rep2:Year1	13.828	0.580	4.337	c
Rep2:Year2	15.079	0.612	4.577	a
Rep3:Year1	13.866	0.574	4.292	c
Rep3:Year2	14.838	0.600	4.493	b

Table S64: **Repetitions Comparison**

Rep	Means	Standard Error	Standard Dev.	Groups
1	14.360	0.408	4.315	b
2	14.453	0.424	4.483	a
3	14.352	0.416	4.401	b

Table S65: **Years Comparison**

Year	Means	Standard Error	Standard Dev.	Groups
1	13.847	0.329	4.263	b
2	14.929	0.344	4.455	a

Table S66: **Drought treatments Comparison**

Drought treatment	Means	Standard Error	Standard Dev.	Groups
10%	19.857	0.429	3.932	a
30%	15.453	0.174	1.599	b
50%	12.232	0.169	1.551	c
70%	10.011	0.145	1.326	d

```
## The minimun for Drought treatments x Years Comparison  is: 70%:Year1 = 9.63.
## The maximum is: 10%:Year2 = 20.46.
```

Table S67: **Drought treatments x Years Comparison**

DroughtxYear Interaction	Means	Standard Error	Standard Dev.	Groups
10%:Year1	19.255	0.597	3.871	b
10%:Year2	20.459	0.609	3.945	a
30%:Year1	14.688	0.218	1.412	d
30%:Year2	16.218	0.217	1.408	c
50%:Year1	11.815	0.233	1.507	f
50%:Year2	12.649	0.231	1.498	e
70%:Year1	9.631	0.199	1.290	h
70%:Year2	10.391	0.195	1.264	g

Table S68: **Cultivars Comparison**

Cultivar	Means	Standard Error	Standard Dev.	Groups
Aidin	14.695	0.927	4.539	f
Araz	14.775	0.836	4.095	f
Eskandar	16.302	0.886	4.341	c
GN15(Garnem)	17.126	1.052	5.154	a
Mamaei	11.446	0.619	3.032	j
Rabie	12.030	0.489	2.398	i
Saba	14.296	0.770	3.774	g
Shahrood10	15.530	0.992	4.859	e
Shahrood12	16.893	1.154	5.652	b
Shahrood13	12.158	0.522	2.556	i
Shahrood21	12.859	0.622	3.045	h
Shahrood6	12.881	0.636	3.118	h
Shahrood7	14.400	0.598	2.928	g
Shahrood8	16.046	1.140	5.586	d

```
## The minimun for Drought treatments x Genoytpes Comparison is: 70%:Mamaei = 7.65.
## The maximum is: 10%:Shahrood12 = 26.08.
```

Table S69: **Drought treatments x Cultivars Comparison**

DroughtxCultivar Interaction	Means	Standard Error	Standard Dev.	Groups
10%:Aidin	21.332	0.300	0.734	d
10%:Araz	20.120	0.284	0.695	e
10%:Eskandar	22.190	0.285	0.699	c
10%:GN15(Garnem)	25.180	0.341	0.836	b
10%:Mamaei	15.213	0.293	0.718	no
10%:Rabie	14.452	0.232	0.569	q
10%:Saba	18.793	0.246	0.602	f
10%:Shahrood10	22.570	0.291	0.714	c
10%:Shahrood12	26.078	0.301	0.738	a
10%:Shahrood13	15.487	0.262	0.641	mn

DroughtxCultivar Interaction	Means	Standard Error	Standard Dev.	Groups
10%:Shahrood21	16.398	0.297	0.728	ijk
10%:Shahrood6	16.260	0.290	0.711	jk
10%:Shahrood7	18.730	0.389	0.954	f
10%:Shahrood8	25.193	0.287	0.704	b
30%:Aidin	15.773	0.348	0.852	lm
30%:Araz	16.672	0.361	0.885	hij
30%:Eskandar	17.600	0.394	0.966	g
30%:GN15(Garnem)	16.885	0.350	0.857	h
30%:Mamaei	13.073	0.346	0.848	stu
30%:Rabie	13.973	0.347	0.849	r
30%:Saba	16.760	0.328	0.804	hi
30%:Shahrood10	16.988	0.356	0.871	h
30%:Shahrood12	16.138	0.352	0.861	kl
30%:Shahrood13	13.487	0.346	0.847	s
30%:Shahrood21	14.530	0.355	0.871	pq
30%:Shahrood6	14.955	0.364	0.891	op
30%:Shahrood7	14.693	0.339	0.830	pq
30%:Shahrood8	14.815	0.365	0.893	opq
50%:Aidin	11.982	0.208	0.510	vw
50%:Araz	12.655	0.200	0.490	u
50%:Eskandar	14.768	0.210	0.514	opq
50%:GN15(Garnem)	14.775	0.196	0.481	opq
50%:Mamaei	9.852	0.210	0.515	z
50%:Rabie	10.675	0.213	0.521	y
50%:Saba	12.005	0.226	0.555	vw
50%:Shahrood10	12.005	0.292	0.714	vw
50%:Shahrood12	13.165	0.223	0.546	st
50%:Shahrood13	9.742	0.230	0.564	z
50%:Shahrood21	11.865	0.211	0.517	vw
50%:Shahrood6	11.768	0.217	0.531	vw
50%:Shahrood7	12.992	0.203	0.497	tu
50%:Shahrood8	13.002	0.215	0.527	tu
70%:Aidin	9.695	0.172	0.421	z
70%:Araz	9.655	0.176	0.432	z
70%:Eskandar	10.648	0.177	0.434	y
70%:GN15(Garnem)	11.665	0.178	0.436	w
70%:Mamaei	7.645	0.178	0.436	C
70%:Rabie	9.022	0.290	0.710	A
70%:Saba	9.625	0.174	0.427	z
70%:Shahrood10	10.555	0.176	0.431	y
70%:Shahrood12	12.188	0.181	0.442	v
70%:Shahrood13	9.918	0.168	0.413	z
70%:Shahrood21	8.642	0.173	0.424	AB
70%:Shahrood6	8.542	0.174	0.427	B
70%:Shahrood7	11.183	0.175	0.429	x
70%:Shahrood8	11.175	0.168	0.410	x

POX MODEL - TWO YEARS

Table S70: Analysis of Variance Total POX

Source of Var.	Df	Sum sq	Mean sq	F value	P value	Result
**RepxYear**	2	7.861	3.93	18.01	6.128e-08	p < .001
**Year**	1	20.63	20.63	94.54	1.169e-18	p < .001
**Rep**	2	0.5944	0.2972	1.362	0.2585	p = 0.259
**Drought**	3	432.3	144.1	660.2	5.547e-106	p < .001
**DroughtxYear**	3	0.2954	0.09845	0.4511	0.7168	p = 0.717
**Error__a**	12	4.464	0.372			
**Cultivar**	13	801.8	61.67	282.6	2.518e-124	p < .001
**CultivarxYear**	13	0.165	0.01269	0.05814	1	p > .999
**DroughtxCultivar**	39	185.7	4.761	21.81	2.219e-54	p < .001
**DroughtxCultivarxYear**	39	1.156	0.02965	0.1358	1	p > .999
**Error__b**	208	45.4	0.2183			
**Total**	335	1500				

```
## [1] "R Square 0.97"
```

```
## [1] "CV(a): 3.169 , CV(b) : 1.739"
```

Table S71: **Other Statistics**

	Standar Error of mean	Standar Error of difference	CD	CD1
Year	0.058	0.082	0.166	0.223
Rep	0.071	0.101	0.204	0.273
Drought	0.082	0.116	0.235	0.315
DroughtxYear	0.116	0.164	0.333	0.445
Cultivar	0.154	0.218	0.440	0.589
CultivarxYear	0.218	0.308	0.622	0.833
DroughtxCultivar	0.308	0.435	0.880	1.178
DroughtxCultivarxYear	0.435	0.615	1.244	1.666

```
## The minimun for Repetitions x Year Comparison is: Rep1:Year1 = 9.49.
## The maximum is: Rep1:Year2 = 10.38.
```

Table S72: **Repetitions x Years Comparison**

RepetitionxYear Interaction	Means	Standard Error	Standard Dev.	Groups
Rep1:Year1	9.486	0.286	2.138	d
Rep1:Year2	10.378	0.281	2.100	a
Rep2:Year1	9.703	0.276	2.062	c
Rep2:Year2	10.151	0.290	2.170	b
Rep3:Year1	9.767	0.287	2.148	c
Rep3:Year2	9.914	0.274	2.048	c

Table S73: **Years Comparison**

Year	Means	Standard Error	Standard Dev.	Groups
1	9.652	0.163	2.107	b
2	10.148	0.162	2.102	a

Table S74: **Drought treatments Comparison**

Drought treatment	Means	Standard Error	Standard Dev.	Groups
10%	11.588	0.233	2.136	a
30%	10.244	0.189	1.732	b
50%	9.118	0.160	1.464	c
70%	8.649	0.194	1.778	d

Table S75: **Cultivars Comparison**

Cultivar	Means	Standard Error	Standard Dev.	Groups
Aidin	10.825	0.304	1.491	c
Araz	7.645	0.235	1.149	g
Eskandar	9.573	0.274	1.344	e
GN15(Garnem)	9.996	0.613	3.003	d
Mamaei	10.560	0.110	0.539	c
Rabie	7.402	0.188	0.919	gh
Saba	10.767	0.251	1.230	c
Shahrood10	11.228	0.366	1.791	b
Shahrood12	8.677	0.302	1.481	f
Shahrood13	9.819	0.135	0.662	de
Shahrood21	12.021	0.146	0.714	a
Shahrood6	11.503	0.199	0.974	b
Shahrood7	7.188	0.198	0.970	h
Shahrood8	11.395	0.462	2.262	b

```
## The minimun for Drought treatments x Genoytpes Comparison is: 70%:Araz = 6.18.
## The maximum is: 10%:GN15(Garnem) = 14.7.
```

Table S76: **Drought treatments x Cultivars Comparison**

DroughtxCultivar Interaction	Means	Standard Error	Standard Dev.	Groups
10%:Aidin	12.415	0.574	1.406	bcd
10%:Araz	9.133	0.166	0.407	lmnop
10%:Eskandar	11.658	0.164	0.402	defg
10%:GN15(Garnem)	14.698	0.153	0.375	a
10%:Mamaei	11.202	0.200	0.490	efgh
10%:Rabie	7.859	0.237	0.580	qrs
10%:Saba	12.462	0.153	0.375	bcd
10%:Shahrood10	13.128	0.769	1.885	b
10%:Shahrood12	10.458	0.200	0.491	hij
10%:Shahrood13	10.245	0.209	0.513	hijk

DroughtxCultivar Interaction	Means	Standard Error	Standard Dev.	Groups
10%:Shahrood21	13.068	0.174	0.427	b
10%:Shahrood6	12.798	0.151	0.369	bc
10%:Shahrood7	8.702	0.153	0.375	mnopq
10%:Shahrood8	14.408	0.158	0.387	a
30%:Aidin	11.688	0.149	0.366	defg
30%:Araz	8.032	0.156	0.383	qr
30%:Eskandar	9.585	0.149	0.366	jklmn
30%:GN15(Garnem)	10.153	0.151	0.370	ijkl
30%:Mamaei	10.500	0.160	0.392	hij
30%:Rabie	7.708	0.151	0.370	qrst
30%:Saba	11.208	0.151	0.370	efgh
30%:Shahrood10	12.275	0.160	0.392	bcd
30%:Shahrood12	9.365	0.162	0.398	klmno
30%:Shahrood13	10.005	0.189	0.464	ijkl
30%:Shahrood21	11.728	0.155	0.380	def
30%:Shahrood6	11.862	0.162	0.398	cde
30%:Shahrood7	6.972	0.153	0.375	stuv
30%:Shahrood8	12.338	0.170	0.417	bcd
50%:Aidin	9.925	0.139	0.341	ijkl
50%:Araz	7.232	0.146	0.357	rstu
50%:Eskandar	8.632	0.117	0.287	nopq
50%:GN15(Garnem)	7.912	0.147	0.359	qrs
50%:Mamaei	10.265	0.156	0.382	hijk
50%:Rabie	7.372	0.142	0.347	rstu
50%:Saba	9.548	0.171	0.419	jklmn
50%:Shahrood10	9.825	0.119	0.292	ijkl
50%:Shahrood12	8.162	0.221	0.542	pqr
50%:Shahrood13	9.675	0.145	0.354	ijklm
50%:Shahrood21	11.575	0.159	0.388	defg
50%:Shahrood6	10.652	0.190	0.466	ghi
50%:Shahrood7	6.645	0.160	0.391	uv
50%:Shahrood8	10.232	0.170	0.417	hijk
70%:Aidin	9.272	0.185	0.452	klmno
70%:Araz	6.183	0.071	0.175	v
70%:Eskandar	8.417	0.090	0.220	opq
70%:GN15(Garnem)	7.220	0.080	0.197	rstuv
70%:Mamaei	10.272	0.124	0.305	hijk
70%:Rabie	6.670	0.617	1.512	tuv
70%:Saba	9.850	0.078	0.192	ijkl
70%:Shahrood10	9.683	0.126	0.308	ijklm
70%:Shahrood12	6.722	0.135	0.331	tuv
70%:Shahrood13	9.350	0.379	0.929	klmno
70%:Shahrood21	11.712	0.126	0.310	def
70%:Shahrood6	10.700	0.084	0.206	fghi
70%:Shahrood7	6.433	0.094	0.230	uv
70%:Shahrood8	8.600	0.108	0.264	nopq

SOD MODEL - TWO YEARS

Table S77: Analysis of Variance Total SOD

Source of Var.	Df	Sum sq	Mean sq	F value	P value	Result
**RepxYear**	2	10.83	5.417	53.42	1.888e-19	p < .001
**Year**	1	0.2227	0.2227	2.196	0.1399	p = 0.140
**Rep**	2	11.5	5.752	56.73	2.17e-20	p < .001
**Drought**	3	2512	837.3	8258	6.139e-216	p < .001
**DroughtxYear**	3	2.202	0.7341	7.24	0.000121	p < .001
**Error__a**	12	3.27	0.2725			
**Cultivar**	13	6321	486.2	4796	9.341e-250	p < .001
**CultivarxYear**	13	0.3651	0.02809	0.277	0.9943	p = 0.994
**DroughtxCultivar**	39	1051	26.94	265.7	9.958e-157	p < .001
**DroughtxCultivarxYear**	39	3.201	0.08206	0.8094	0.7817	p = 0.782
**Error__b**	208	21.09	0.1014			
**Total**	335	9936				

```
## [1] "R Square 0.998"
```

```
## [1] "CV(a): 4.453 , CV(b) : 1.489"
```

Table S78: **Other Statistics**

	Standar Error of mean	Standar Error of difference	CD	CD1
Year	0.058	0.082	0.166	0.223
Rep	0.071	0.101	0.204	0.273
Drought	0.082	0.116	0.235	0.315
DroughtxYear	0.116	0.164	0.333	0.445
Cultivar	0.154	0.218	0.440	0.589
CultivarxYear	0.218	0.308	0.622	0.833
DroughtxCultivar	0.308	0.435	0.880	1.178
DroughtxCultivarxYear	0.435	0.615	1.244	1.666

```
## The minimun for Repetitions x Year Comparison is: Rep1:Year1 = 18.75.
## The maximum is: Rep3:Year1 = 19.56.
```

Table S79: **Repetitions x Years Comparison**

RepetitionxYear Interaction	Means	Standard Error	Standard Dev.	Groups
Rep1:Year1	18.752	0.737	5.513	c
Rep1:Year2	19.208	0.733	5.484	b
Rep2:Year1	19.488	0.737	5.518	a
Rep2:Year2	19.199	0.731	5.467	b
Rep3:Year1	19.556	0.737	5.515	a
Rep3:Year2	19.235	0.720	5.389	b

Table S80: **Repetitions Comparison**

Rep	Means	Standard Error	Standard Dev.	Groups
1	18.980	0.518	5.478	b
2	19.344	0.517	5.470	a
3	19.396	0.513	5.430	a

Table S81: **Drought treatments Comparison**

Drought treatment	Means	Standard Error	Standard Dev.	Groups
10%	23.465	0.753	6.903	a
30%	19.624	0.509	4.665	b
50%	17.732	0.386	3.540	c
70%	16.137	0.299	2.740	d

```
## The minimun for Drought treatments x Years Comparison  is: 70%:Year1 = 16.03.
## The maximum is: 10%:Year1 = 23.5.
```

Table S82: **Drought treatments x Years Comparison**

DroughtxYear Interaction	Means	Standard Error	Standard Dev.	Groups
10%:Year1	23.504	1.078	6.985	a
10%:Year2	23.427	1.065	6.904	a
30%:Year1	19.690	0.725	4.698	b
30%:Year2	19.559	0.723	4.689	b
50%:Year1	17.839	0.550	3.561	c
50%:Year2	17.625	0.549	3.559	d
70%:Year1	16.029	0.437	2.832	f
70%:Year2	16.245	0.413	2.674	e

Table S83: **Cultivars Comparison**

Cultivar	Means	Standard Error	Standard Dev.	Groups
Aidin	19.542	0.538	2.636	f
Araz	17.147	0.630	3.088	g
Eskandar	21.969	0.771	3.777	e
GN15(Garnem)	26.943	1.255	6.150	a
Mamaei	17.076	0.232	1.134	g
Rabie	17.062	0.234	1.148	g
Saba	12.796	0.400	1.958	k
Shahrood10	23.698	0.927	4.542	d
Shahrood12	25.445	0.995	4.876	b
Shahrood13	14.949	0.326	1.596	j
Shahrood21	15.665	0.317	1.553	i
Shahrood6	15.544	0.283	1.387	i
Shahrood7	16.677	0.241	1.178	h
Shahrood8	24.840	1.109	5.431	c

```
## The minimun for Drought treatments x Genoytpes Comparison is: 70%:Saba = 10.92.
## The maximum is: 10%:GN15(Garnem) = 35.35.
```

Table S84: **Drought treatments x Cultivars Comparison**

DroughtxCultivar Interaction	Means	Standard Error	Standard Dev.	Groups
10%:Aidin	23.442	0.162	0.397	g
10%:Araz	22.150	0.163	0.400	hi
10%:Eskandar	27.257	0.212	0.520	e
10%:GN15(Garnem)	35.350	0.237	0.581	a
10%:Mamaei	18.770	0.154	0.377	mn
10%:Rabie	18.363	0.182	0.447	no
10%:Saba	15.845	0.357	0.876	xy
10%:Shahrood10	30.897	0.168	0.412	c
10%:Shahrood12	33.160	0.173	0.424	b
10%:Shahrood13	16.713	0.324	0.794	stuv
10%:Shahrood21	17.670	0.118	0.289	pq
10%:Shahrood6	17.070	0.134	0.328	qrst
10%:Shahrood7	18.455	0.271	0.664	n
10%:Shahrood8	33.370	0.151	0.370	b
30%:Aidin	20.145	0.125	0.305	jk
30%:Araz	16.135	0.122	0.300	vwx
30%:Eskandar	23.352	0.104	0.254	g
30%:GN15(Garnem)	29.273	0.201	0.493	d
30%:Mamaei	17.092	0.122	0.298	qrst
30%:Rabie	17.333	0.129	0.316	pqrs
30%:Saba	12.648	0.111	0.272	C
30%:Shahrood10	23.285	0.113	0.278	g
30%:Shahrood12	25.395	0.159	0.390	f
30%:Shahrood13	15.387	0.120	0.294	yz
30%:Shahrood21	16.510	0.153	0.375	tuvw
30%:Shahrood6	16.660	0.126	0.308	tuv
30%:Shahrood7	16.628	0.111	0.273	tuv
30%:Shahrood8	24.898	0.222	0.545	f
50%:Aidin	17.775	0.211	0.518	op
50%:Araz	16.195	0.138	0.338	uvwx
50%:Eskandar	19.675	0.113	0.277	kl
50%:GN15(Garnem)	23.888	0.097	0.238	g
50%:Mamaei	16.548	0.146	0.358	tuvw
50%:Rabie	16.947	0.145	0.355	rst
50%:Saba	11.773	0.122	0.299	D
50%:Shahrood10	21.682	0.112	0.275	i
50%:Shahrood12	22.577	0.140	0.342	h
50%:Shahrood13	14.780	0.131	0.320	zA
50%:Shahrood21	14.465	0.157	0.384	AB
50%:Shahrood6	14.132	0.137	0.335	AB
50%:Shahrood7	16.057	0.123	0.301	vwx
50%:Shahrood8	21.757	0.148	0.363	i
70%:Aidin	16.808	0.082	0.201	stu
70%:Araz	14.108	0.100	0.246	B
70%:Eskandar	17.592	0.087	0.214	pqr
70%:GN15(Garnem)	19.262	0.078	0.192	lm
70%:Mamaei	15.893	0.131	0.321	wxy

DroughtxCultivar Interaction	Means	Standard Error	Standard Dev.	Groups
70%:Rabie	15.603	0.396	0.971	xy
70%:Saba	10.917	0.072	0.177	E
70%:Shahrood10	18.930	0.081	0.198	mn
70%:Shahrood12	20.650	0.064	0.157	j
70%:Shahrood13	12.917	0.568	1.391	C
70%:Shahrood21	14.013	0.079	0.195	B
70%:Shahrood6	14.315	0.083	0.202	AB
70%:Shahrood7	15.570	0.093	0.229	xy
70%:Shahrood8	19.337	0.092	0.226	lm

GPX MODEL - TWO YEARS

Table S85: Analysis of Variance Total GPX

Source of Var.	Df	Sum sq	Mean sq	F value	P value	Result
**RepxYear**	2	2.143	1.071	13.76	2.434e-06	p < .001
**Year**	1	10.61	10.61	136.3	1.513e-24	p < .001
**Rep**	2	1.465	0.7324	9.41	0.0001223	p < .001
**Drought**	3	4565	1522	19550	1.207e-254	p < .001
**DroughtxYear**	3	12.77	4.256	54.68	4.225e-26	p < .001
**Error_a**	12	6.736	0.5613			
**Cultivar**	13	7569	582.3	7481	8.78e-270	p < .001
**CultivarxYear**	13	0.3208	0.02468	0.3171	0.989	p = 0.989
**DroughtxCultivar**	39	852.2	21.85	280.7	3.709e-159	p < .001
**DroughtxCultivarxYear**	39	0.9783	0.02509	0.3223	1	p > .999
**Error_b**	208	16.19	0.07783			
**Total**	335	13038				

```
## [1] "R Square 0.999"
```

```
## [1] "CV(a): 12.064 , CV(b) : 0.926"
```

Table S86: **Other Statistics**

	Standar Error of mean	Standar Error of difference	CD	CD1
Year	0.058	0.082	0.166	0.223
Rep	0.071	0.101	0.204	0.273
Drought	0.082	0.116	0.235	0.315
DroughtxYear	0.116	0.164	0.333	0.445
Cultivar	0.154	0.218	0.440	0.589
CultivarxYear	0.218	0.308	0.622	0.833
DroughtxCultivar	0.308	0.435	0.880	1.178
DroughtxCultivarxYear	0.435	0.615	1.244	1.666

```
## The minimun for Repetitions x Year Comparison is: Rep1:Year1 = 16.82.
## The maximum is: Rep1:Year2 = 17.39.
```

Table S87: **Repetitions x Years Comparison**

RepetitionxYear Interaction	Means	Standard Error	Standard Dev.	Groups
Rep1:Year1	16.817	0.826	6.181	d
Rep1:Year2	17.393	0.862	6.453	a
Rep2:Year1	16.915	0.813	6.082	cd
Rep2:Year2	17.119	0.834	6.239	b
Rep3:Year1	17.036	0.852	6.374	bc
Rep3:Year2	17.321	0.849	6.356	a

Table S88: **Repetitions Comparison**

Rep	Means	Standard Error	Standard Dev.	Groups
1	17.105	0.595	6.297	b
2	17.017	0.580	6.134	c
3	17.178	0.599	6.338	a

Table S89: **Years Comparison**

Year	Means	Standard Error	Standard Dev.	Groups
1	16.922	0.477	6.177	b
2	17.278	0.487	6.313	a

Table S90: **Drought treatments Comparison**

Drought treatment	Means	Standard Error	Standard Dev.	Groups
10%	23.048	0.798	7.315	a
30%	17.159	0.485	4.443	b
50%	14.775	0.434	3.981	c
70%	13.418	0.393	3.602	d

```
## The minimun for Drought treatments x Years Comparison  is: 70%:Year2 = 13.41.
## The maximum is: 10%:Year2 = 23.34.
```

Table S91: **Drought treatments x Years Comparison**

DroughtxYear Interaction	Means	Standard Error	Standard Dev.	Groups
10%:Year1	22.756	1.133	7.342	b
10%:Year2	23.340	1.137	7.367	a
30%:Year1	16.719	0.690	4.469	d
30%:Year2	17.598	0.683	4.426	c
50%:Year1	14.789	0.618	4.006	e
50%:Year2	14.762	0.618	4.006	e
70%:Year1	13.425	0.564	3.658	f
70%:Year2	13.411	0.554	3.590	f

Table S92: **Cultivars Comparison**

Cultivar	Means	Standard Error	Standard Dev.	Groups
Aidin	18.027	0.873	4.277	f
Araz	18.088	0.818	4.007	f
Eskandar	18.455	0.873	4.277	e
GN15(Garnem)	24.819	1.252	6.133	a
Mamaei	11.028	0.431	2.110	k
Rabie	9.910	0.341	1.672	l
Saba	17.821	0.757	3.710	g
Shahrood10	20.152	0.991	4.853	d
Shahrood12	22.413	1.194	5.851	c
Shahrood13	11.175	0.317	1.551	k
Shahrood21	11.877	0.623	3.053	j
Shahrood6	14.341	0.507	2.483	i
Shahrood7	16.753	0.736	3.608	h
Shahrood8	24.542	1.255	6.147	b

```
## The minimun for Drought treatments x Genoytpes Comparison is: 70%:Rabie = 8.45.
## The maximum is: 10%:GN15(Garnem) = 34.5.
```

Table S93: **Drought treatments x Cultivars Comparison**

DroughtxCultivar Interaction	Means	Standard Error	Standard Dev.	Groups
10%:Aidin	24.452	0.170	0.416	e
10%:Araz	24.417	0.239	0.584	e
10%:Eskandar	25.075	0.309	0.758	d
10%:GN15(Garnem)	34.505	0.219	0.537	a
10%:Mamaei	14.305	0.260	0.636	uv
10%:Rabie	12.515	0.138	0.337	zA
10%:Saba	23.137	0.175	0.429	g
10%:Shahrood10	28.098	0.239	0.585	c
10%:Shahrood12	32.232	0.277	0.677	b
10%:Shahrood13	13.158	0.124	0.304	xy
10%:Shahrood21	16.302	0.333	0.815	p
10%:Shahrood6	18.095	0.240	0.588	no
10%:Shahrood7	21.885	0.162	0.397	h
10%:Shahrood8	34.498	0.136	0.334	a
30%:Aidin	18.913	0.292	0.716	kl
30%:Araz	18.203	0.198	0.485	mno
30%:Eskandar	18.552	0.189	0.464	klmn
30%:GN15(Garnem)	24.947	0.199	0.486	de
30%:Mamaei	11.098	0.264	0.646	C
30%:Rabie	9.840	0.285	0.697	DE
30%:Saba	18.732	0.213	0.521	klm
30%:Shahrood10	18.738	0.261	0.640	klm
30%:Shahrood12	20.273	0.244	0.598	j
30%:Shahrood13	11.998	0.244	0.598	AB
30%:Shahrood21	12.738	0.249	0.611	yz
30%:Shahrood6	14.633	0.201	0.493	tu
30%:Shahrood7	17.738	0.264	0.648	o

DroughtxCultivar Interaction	Means	Standard Error	Standard Dev.	Groups
30%:Shahrood8	23.815	0.253	0.620	f
50%:Aidin	14.930	0.165	0.404	st
50%:Araz	15.203	0.081	0.199	rs
50%:Eskandar	16.313	0.098	0.239	p
50%:GN15(Garnem)	20.833	0.095	0.233	ij
50%:Mamaei	9.420	0.045	0.111	E
50%:Rabie	8.833	0.073	0.179	FG
50%:Saba	16.103	0.059	0.144	pq
50%:Shahrood10	18.137	0.047	0.114	no
50%:Shahrood12	18.407	0.043	0.106	lmn
50%:Shahrood13	10.030	0.024	0.059	D
50%:Shahrood21	9.770	0.046	0.113	DE
50%:Shahrood6	12.927	0.049	0.119	xyz
50%:Shahrood7	14.983	0.084	0.206	st
50%:Shahrood8	20.967	0.059	0.145	i
70%:Aidin	13.812	0.021	0.052	vw
70%:Araz	14.530	0.025	0.061	tu
70%:Eskandar	13.880	0.107	0.263	v
70%:GN15(Garnem)	18.990	0.040	0.098	k
70%:Mamaei	9.287	0.043	0.107	EF
70%:Rabie	8.450	0.146	0.357	G
70%:Saba	13.313	0.043	0.105	wx
70%:Shahrood10	15.635	0.083	0.204	qr
70%:Shahrood12	18.740	0.023	0.057	klm
70%:Shahrood13	9.513	0.182	0.445	DE
70%:Shahrood21	8.700	0.036	0.088	G
70%:Shahrood6	11.710	0.068	0.168	B
70%:Shahrood7	12.403	0.065	0.160	zA
70%:Shahrood8	18.890	0.073	0.179	kl
